# Fecal microbiota transplantation and bacterial consortium transplantation have comparable effects on the re-establishment of mucosal barrier function in mice with intestinal dysbiosis

**DOI:** 10.3389/fmicb.2015.00692

**Published:** 2015-07-07

**Authors:** Ming Li, Pin Liang, Zhenzhen Li, Ying Wang, Guobin Zhang, Hongwei Gao, Shu Wen, Li Tang

**Affiliations:** ^1^Department of Microecology, School of Basic Medical Science, Dalian Medical UniversityDalian, China; ^2^Key Microecology Laboratory of Liaoning ProvinceDalian, China; ^3^Department of General Surgery, The First Affiliated Hospital of Dalian Medical UniversityDalian, China

**Keywords:** intestinal dysbiosis, fecal microbiota transplantation, bacterial consortia transplantation, mucosal barrier function, intestinal microbiota

## Abstract

Fecal microbiota transplantation (FMT) is a promising therapy, despite some reports of adverse side effects. Bacterial consortia transplantation (BCT) for targeted restoration of the intestinal ecosystem is considered a relatively safe and simple procedure. However, no systematic research has assessed the effects of FMT and BCT on immune responses of intestinal mucosal barrier in patients. We conducted complementary studies in animal models on the effects of FMT and BCT, and provide recommendations for improving the clinical outcomes of these treatments. To establish the dysbiosis model, male BALB/c mice were treated with ceftriaxone intra-gastrically for 7 days. After that, FMT and BCT were performed on ceftriaxone-treated mice for 3 consecutive days to rebuild the intestinal ecosystem. Post-FMT and post-BCT changes of the intestinal microbial community and mucosal barrier functions were investigated and compared. Disruption of intestinal microbial homeostasis impacted the integrity of mucosal epithelial layer, resulting in increased intestinal permeability. These outcomes were accompanied by overexpression of Muc2, significant decrease of SIgA secretion, and overproduction of defensins and inflammatory cytokines. After FMT and BCT, the intestinal microbiota recovered quickly, this was associated with better reconstruction of mucosal barriers and re-establishment of immune networks compared with spontaneous recovery (SR). Although based on a short-term study, our results suggest that FMT and BCT promote the re-establishment of intestinal microbial communities in mice with antibiotic-induced dysbiosis, and contribute to the temporal and spatial interactions between microbiota and mucosal barriers. The effects of BCT are comparable to that of FMT, especially in normalizing the intestinal levels of Muc2, SIgA, and defensins.

## Introduction

Commensal bacteria living in the human gastrointestinal tract provide a biological barrier against the invasion of pathogens and contribute to the modulation of the immune system (Hooper et al., 2012; Maynard et al., 2012). Disturbance of intestinal microbiota may result in dysfunction of the mucosal barrier, as it has been shown in animal models that treatment of antibiotics altered the colonic mucus layer (Wlodarska et al., 2011), increased bacterial translocation (Wang et al., 2014), and led to intestinal sensory and motor changes (Aguilera et al., 2015). Intestinal microbes have been proved to affect mucin gene expression (Becker et al., 2013), mucosal permeability, secretion of secretory immunoglobulin A (SIgA) and antibacterial peptides, as well as of various cytokines by cells on mucosal surfaces (Lutgendorff et al., 2008). Dysfunction of the intestinal barrier increases risk for infection and metabolic disorders, which are closely related to a variety of gastrointestinal diseases (Turner, 2009). Re-establishing the equilibrium of the intestinal microbiota is therefore important for clinical improvement in patients suffering from gastrointestinal diseases.

The use of probiotics can restore function to a disrupted mucosal barrier (Dicksved et al., 2012). However, in patients with intestinal dysbiosis—significant qualitative and quantitative changes in the intestinal microbiota (Hawrelak and Myers, 2004)—it can be difficult to completely eradicate harmful bacteria, and selectively restore and maintain a community of healthy bacteria. Recently, fecal microbiota transplantation (FMT) has received much public attention (van Nood et al., 2013). Because of the high cure rate (over 90%) and rare occurrence of side effects, FMT has been considered a potentially life-saving “last chance” option for the treatment of recurrent *Clostridium difficile* infection (Bakken et al., 2011). This has generated interest in applying this therapy to the treatment of other gastrointestinal diseases. Several studies have reported that infusion of fecal suspension from healthy individuals to inflammatory bowel disease (IBD) (Bennet and Brinkman, 1989; Kunde et al., 2013; Kao et al., 2014) or irritable bowel syndrome (IBS) (Konig and Brummer, 2014; Shanahan and Quigley, 2014) patients resulted in clinical improvement and remission. It has been suggested by many scientists that FMT has promising therapeutic potential for the treatment of diabetes (Udayappan et al., 2014), obesity, non-alcoholic fatty liver disease (NAFLD), and even cardiovascular disease (Smits et al., 2013). However, some studies have reported less than ideal outcomes of FMT (Vermeire et al., 2012; Kump et al., 2013), and significant differences have been found regarding the colonization of bacteria in the gastrointestinal tract of different patients (Angelberger et al., 2013). Some researchers have attempted mixed bacterial consortium transplantation (BCT) to re-establish the intestinal ecosystem in animal models of digestive and metabolic diseases (Lawley et al., 2012). This method is considered to have better control and to be relatively stable (Petrof and Khoruts, 2014), and thus is worthy of in-depth study. However, no systematic research has assessed the effects of FMT/BCT on immune responses of the intestinal mucosal barrier in patients. In fact, colonization by donor bacteria and changes of mucosal physiology in the human intestine can be hard to achieve. Complementary studies of post-FMT or post-BCT changes in animal models are important to improve clinical efficacy.

In this study, we induced intestinal dysbiosis in BALB/c mice by using the broad-spectrum antibiotic ceftriaxone sodium, which is widely used in China for the treatment of infections in the respiratory and digestive tracts. Two bacteriotherapies, FMT and BCT, were adopted to re-establish intestinal microbial equilibrium. Post-FMT and post-BCT changes of the gut mucosal barrier function in mouse models were investigated and compared. This study helps our understanding of the effects of intestinal microbiota on mucosal barrier function during occurrence, development, and prognosis of diseases, and provides new insight into the development of microbiota transplantation therapy.

## Materials and methods

### Animals

Male BALB/c mice (aged 6–8 weeks, weighing 18 ± 22 g) were provided by the Experimental Animal House of Dalian Medical University, where they were maintained under specific pathogen-free conditions (light/dark cycles of 12 h). All mice of the same experimental conditions were co-housed in a filter top cage, and given food and water *ad libitum*. To establish the dysbiosis model, mice were treated with 0.2 mL ceftriaxone sodium (400 mg/mL) intra-gastrically twice a day with an interval of 6 h for 7 days. For controls, sterile water was used. 30 mice were used in each experimental group (total 120 mice), and they were sacrificed by cervical dislocation after inhalational anesthesia at indicated time point (Please see details of the animal tests in the Figure S1). The animal experimental procedures were approved by the Medical Ethics Committee of Dalian Medical University, China (SYXK2012-0002).

### Transplantation of microbiota

For FMT: Fresh feces was collected from 5 healthy BALB/c mice, homogenized in 10 mL of sterile PBS and centrifuged for 30 s at 3000 r.p.m., 4°C, to pellet the particulate matter. OD value of the supernatant slurry was checked to calculate the concentration of total bacteria (OD = 0.5 represents 10^8^ cells). For each mouse, 1 × 10^9.8^ bacterial cells (sum of total bacterial population within 2 g cecal contents) were centrifuged for 5 min at 12,000 r.p.m., 4°C, the bacterial pellets was re-suspended in 0.5 mL PBS and gavaged into each mouse 1 day at a time.

For BCT: Individual bacteria isolated from mouse feces were grown in different liquid medium (see Table 1) for 12–48 h under aerobic or anaerobic conditions at 37°C. Bacteria were harvested by centrifugation and re-suspended in sterile PBS. The density of each bacterium was measured through OD detection, according to the population in mouse feces (Table 1), different volumes of bacteria were calculated and mixed to form the bacterial consortium, all the cells were then centrifuged and re-suspended in 0.5 mL PBS and gavaged into each mouse 1 day at a time.

**Table 1:** **Identification, culture condition, and the population of commensal bacteria in mouse feces**.

**Isolates**	**Strain with highest identity**	**Identity (%)**	**Phylum**	**Gram stain**	**Selective medium^a^**	**Growth condition**		**Abundances (LogCFU/g)**	**Sequences of primers**	**Target bacterial group (s)**	**References**
						**Air condition**	**Growth time (h)**				
DMBCT1	*Bifidobacterium thermophilum* RBL67	98	Actinobacteria	+	BS agar	Anaerobic	48	8.867	BifF: TCGCGTCCGGTGTGAAAG g-Bifid-R: CCACATCCAGCGTCCAC	*Bifidobacterium* spp.	Rinttila et al., 2004
DMBCT2	*Escherichia coli* str. K-12	99	Proteobacteria	−	EMB agar	Aerobic	24	6.193	ECFW: CATGCCGCGTGTATGAAGAA ECRV: CGGGTAACGTCAATGAGCAAA	*Escherichia. coli*	Huijsdens et al., 2002^b^
DMBCT3	*Enterococcus hirae* ATCC 9790	98	Firmicutes	+	EC agar	Anaerobic	24	7.413	ECST748F: AGAAATTCCAAACGAACTTG ENC854R: CAGTGCTCTACCTCCATCATT	*Enterococcus* spp.	Ludwig and Schleifer, 2000^b^
DMBCT4	*Staphylococcus aureus* subsp. NCTC 8325	99	Firmicutes	+	BP agar	Aerobic	24	7.031	STPYF: ACGGTCTTGCTGTCACTTATA STPYR2: TACACATATGTTCTTCCCTAATAA	*Staphylococcus* spp.	Matsuda et al., 2007
DMBCT5	*Fusobacterium nucleatum* subsp. vincentii 3_1_36A2	97	Fusobacteria	−	FS agar	Anaerobic	24	7.251	FusoF: GGATTTATTGGGCGTAAAGC FusoR: GGCATTCCTACAAATATCTACGAA	*Fusobacterium* spp.	Boutaga et al., 2005^b^
DMBCT6	*Lactobacillus reuteri* DSM 20016	99	Firmicutes	+	MRS agar	Anaerobic	12	8.993	LactoF: AGCAGTAGGGAATCTTCCA LactoR: CACCGCTACACATGGAG	*Lactobacillus* group	Walter et al., 2001; Heilig et al., 2002
DMBCT7	*Bacteroidesalanitronis* DSM 18170	94	Bacteroidetes	−	BDS agar	Anaerobic	24	7.703	BactF: GGTGTCGGCTTAAGTGCCAT BactR: CGGA(C/T)GTAAGGGCCGTGC	*Bacteroides-Prevotella-Porphyromonas*	Rinttila et al., 2004
DMBCT8	*Streptococcus thermophilus* CNRZ1066	98	Firmicutes	+	TATAC agar	Anaerobic	24	9.134	Strep-F: AGATGGACCTGCGTTGT Stherm08: GTGAACTTTCCACTCTCACAC	*Streptococcus* spp.	Rudney et al., 2003; Furet et al., 2004
DMBCT9	*Veillonella parvula* DSM 2008	98	Firmicutes	−	VS agar	Anaerobic	24	8.661	Veil-F: ATCAACCTGCCCTTCAGA Veil-R: CGTCCCGATTAACAGAGCTT	*Veillonella* spp.	Rinttila et al., 2004
DMBCT10	*Peptococcus niger* spp.	94	Firmicutes	+	PMS agar	Anaerobic	24	4.148	Pep0830: GGTGCCGCAGTAAACACAATAAG Pep1369: AAGGCCCGGGAACGTATTCA	*Peptococcus* spp.	Rekha et al., 2006
DMBCT11	*Eubacterium siraeum* V10Sc8a	94	Firmicutes	+	ES agar	Anaerobic	48	7.402	EubacF: CGGTACCTGACTAAGAAGC EubacR: AGTTT(C/T)ATTCTTGCGAACG	*Eubacterium rectale-Clostridium coccoides* groups	Rinttila et al., 2004

a *BS, Bifidobacterium selective; EMB, Eosin methylene blue; EC, Enterococcus selective; BP, Blaird-paker; FS, Fusobacterium selective; MRS, deMan Rogosa Sharpe, with Vancomycin, bromocresol green; BDS, Bacteroides selective; TATAC, Triphenyl tetrazolium chloride, acridine orange, thallous sulfate, aesculin, and crystal violet; VS, Veillonella selective; PMS, Peptococcus and Micrococcus selective; ES, Eubacterium selective. The compositions of different selective media were given in Supplementary Materials*.

b *A Taqman probe was used in the original study to evaluate the target bacterial group*.

The control and SR mice were gavaged 0.5 mL PBS 1 day at a time. All mice were treated at the same time and dispatched in different groups.

### Isolation and quantitative real-time PCR (qPCR) analysis of commensal bacteria in mice

For isolation of strains, a mixture of feces from 10 individual healthy mice was used. Feces were diluted with two volumes of sterile phosphate-buffered saline (PBS) and homogenized by vortex. The homogenized solutions were serially diluted in sterile PBS ranging from 10^−1^ to 10^−6^. Diluted suspensions (20 μL) were plated on selective agar media (Table 1) and cultured at 37°C. Single colonies were picked and identified by microscopy and 16S rDNA sequencing; the sequences of the isolates were deposited in NCBI GenBank under the accession number of KM056276 ~ KM056286.

The numbers of 11 selected bacterial groups in cecal contents of mice were quantified using the Thermal Cycler Dice Real Time SystemII (Takara, Japan). Amplification and detection were carried out in 96-well plates using SYBR® Premix DimerEraser™ (Perfect Real Time, Takara, Japan). Each reaction was done in triplicate in a 25 μL total reaction mixture using 2 μL of appropriate dilutions of the DNA sample and 0.3 mM final quantitative PCR primer concentration. The amplification cycle used was one cycle of 95°C for 1 min, followed by 50 cycles of 95°C for 5 s, 55 ~ 68°C for 30 s, 72°C for 30 s, and dissociation at 95°C for 15 s, 60°C for 30 s, and 95°C for 15 s. For construction of the standard curve, the PCR products were generated using the standard PCR primers listed in Table 1 and genomic DNA of different isolates. The copy numbers of samples were determined by reading off the standard series with the Ct values of the samples as described previously (Eeckhaut et al., 2013). Gene copy numbers were expressed as Log10 values per gram wet weight of cecal contents.

### Denaturing gradient gel electrophoresis (DGGE) profiling

The metagenomic DNA was extracted from the frozen cecal content of randomly selected mice (5/group) by the QIAamp DNA stool mini kit (Qiagen, Germany). PCR was conducted using universal primers F338+GC clamp and R518 targeting the hypervariable V3 region of 16S rRNA gene. The resulting 16S rDNA amplicons were analyzed using the DCode system (Bio-Rad, USA) according to descriptions of Joossens et al. (2011). Digitized DGGE images were analyzed with Quantity One image analysis software (version 4.6.1, Bio-Rad USA). To identify specific bands, the DNA extraction and sequencing was performed according to Joossens et al. (2011).

### Collection of biological samples and detection of SIgA, Muc2, defensins, and cytokines

The ileum and colon were removed from mice. Five milli liter of PBS (pH 7.2) containing 0.02% sodium azide was passed through the intestinal segments in order to collect the intestinal mucus. The washout material was centrifuged at 4000 r.p.m. for 20 min at 4°C, the supernatant was harvested, and proteinase inhibitor PMSF was added at final concentration of 1 mmol/L. Intestinal mucus were stored at −80°C for subsequent analysis. Tissues were immediately placed in 10% neutral buffered formalin (Fisher) (48 h, 4°C) for histological studies. Peripheral blood was taken from mice and centrifuged immediately at 1500 × g for 15 min at 4°C to obtain serum. Serum samples were stored at −80°C until analyses. The concentrations of SIgA, Muc2, defensins in intestinal mucus and serum cytokines and IgA were assayed by ELISA (USCN, USA) according to the manufacture's instruction.

### Morphological examination and histological analysis

The tissue samples of the distal ileum and proximal colon were cut into ultra-thin cross sections, and they were fixed in 10% neutral buffered formalin, dehydrated, and paraffin-embedded. The sections were hematoxylin/eosin (HE) stained and examined under a phase-contrast microscope for morphological characteristics. The histological damage was scored using the following criteria: extent of destruction of normal epithelial architecture (0, 1, 2, and 3, for normal, mild, moderate, and extensive damage, respectively); presence and degree of inflammatory infiltration (0, 1, 2, and 3, for normal, mild localized infiltrate, mild generalized infiltrate, and severe generalized infiltrate, respectively); presence of edema (0, 1, 2, and 3, for normal, mild, moderate, and extensive edema, respectively); extent of vascular dilatation and congestion (0, 1, 2, and 3, for normal, mild, moderate, and extensive vascular dilatation and congestion, respectively); presence or absence of goblet cell depletion (0, absent; 1, present), presence or absence of crypt abscesses (0, absent; 1, present). The scores for each criterion were then summed with a maximum possible score of 14 as previously described (Gonzalez-Rey et al., 2006; Aguilera et al., 2015; Grasa et al., 2015). To observe the microvilli, the ultra-thin sections were stained with uranyl acetate and lead citrate, and observed under a JEM-1400 TEM (Olympus, Japan).

### Assessment of gut permeability

Gut permeability was measured as described previously with minor modifications (Wang et al., 2001). The segments of distal ileum and proximal colon were opened along the mesenteric border and mounted in the Ussing chamber with an aperture of 0.3 cm^2^. The chamber was connected to a VCC MC6 amplifier, controlled and monitored using the Acquire and Analyze software (V2.3.177 Physiologic Instruments, San Diego, CA, USA). Experiments were carried out under current-clamp (open-circuit) conditions as described previously (Lee et al., 2007). Segments were incubated in oxygenated (95% O_2_; 5% CO_2_) bicarbonate buffer at 37°C. Tissue was equilibrated for 15 min, thereafter a 3 mA current pulse was applied across the intestinal wall every 6 s for 30 min. The trans-epithelial potential was measured and recorded by the Acquire and Analyze software, and the change in potential induced by the current pulse was used to calculate trans-epithelial resistance (TER) according to Ohm's Law. Higher values of TER represent lower gut permeability.

### Immunohistochemical staining

Four micro miter-thick cross sections of formalin-fixed, paraffin-embedded ileum and colon tissue were cut, deparaffinized, and subjected to a heat-induced epitope retrieval step. Slides were rinsed in cool running water, washed in Tris-buffered saline (pH7.4) before incubation with primary polyclonal antibody against mouse Muc2 (USCN, USA, dilution 1:100) overnight at 4°C. For detection, rabbit anti-rat secondary antibody was used followed by application of the peroxidase kit (USCN, USA). Alkaline phosphatase was revealed by Fast Red as chromogen and peroxidase was developed with a highly sensitive diaminobenzidine (DBA) chromogenic substrate for approximately 10 min. Negative controls were performed by omitting the primary antibody.

### Statistical analysis

The relative position and intensity of DNA bands on DGGE profiles were used for principal component analysis (PCA) by SPSS 16.0 statistical package (SPSS Inc. IL, USA). Statistical analyses were performed using a two-tailed Student *t*-test, with the assistance from GraphPad Prism Program (version 5.01, GraphPad Software Inc., USA). All values were expressed as means ± standard deviation (SD) and the sample sizes. Significance was accepted at *p* < 0.05. If not otherwise specified, statistical significance was indicated as follows: ^*^p < 0.05; ^**^p < 0.01; ^***^p < 0.001.

## Results

### Phenotype of BALB/c mice after ceftriaxone treatment

**Figure 1:**
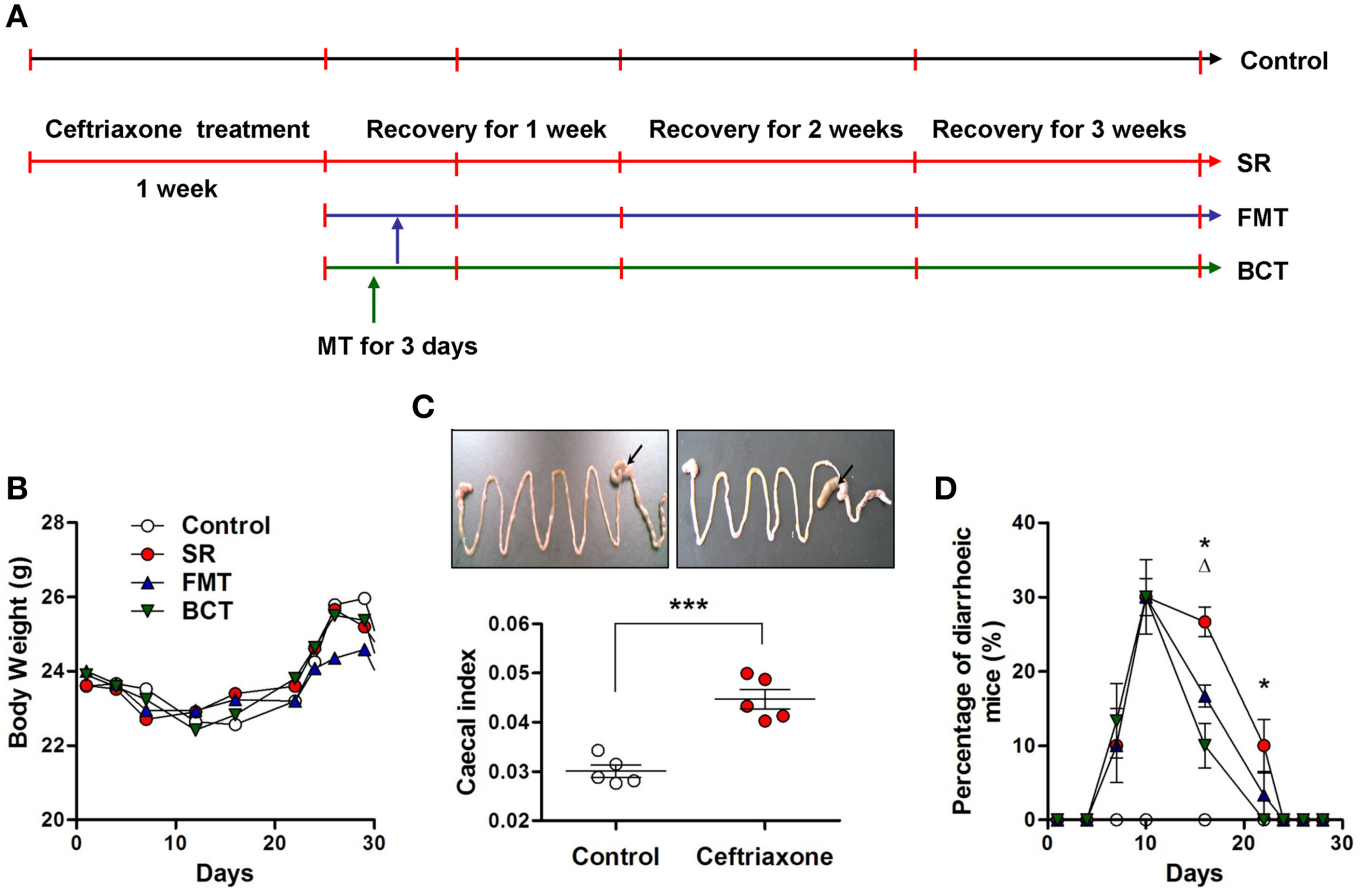
**Schematic overview of ceftriaxone treatment and FMT/BCT. (A)** To establish the intestinal dysbiosis model, BALB/c mice were treated with ceftriaxone sodium for 7 days. After that, they were divided into 3 experimental groups: the spontaneous recovery group (SR), the fecal microbiota transplantation group (FMT), and the bacterial consortium transplantation group (BCT). The red vertical bars indicate sampling dates. The recovery of intestinal ecosystem was observed for 3 weeks. **(B)** Mean body weights, **(C)** Cecal index, and **(D)** The number of mice with diarrhea were investigated respectively. ^***^p < 0.001; ^*^, FMT compared with SR, *p* < 0.05; ^Δ^, BCT compared with FMT, *p* < 0.05.

The experimental design is shown in Figure 1A. During administration of ceftriaxone and over the course of recovery, there were no incidences of mortality, and no significant changes in body weight were observed (Figure 1B). Compared with control mice, the average size of the cecum in ceftriaxone-treated mice was clearly larger (Figure 1C, top), and the ratio of cecal weight to body weight, termed the cecal index, was significantly higher (Figure 1C, *p* = 0.002). After 7 days of ceftriaxone treatment, antibiotic-associated diarrhea was observed in several mice. The number of diarrheic mice did not reduce during the 3 days of transplantation, but instead increased continually. On day 10, the average number of mice with diarrhea in each group was 9, which was 30.00% of the total (Figure 1D). Symptoms of diarrhea subsequently eased such that on day 16, significant differences in the percentage of diarrheic mice were observed between the FMT/BCT groups and the SR group (*p* = 0.0213; *p* = 0.0036), and between the BCT group and the FMT group (*p* = 0.0381).

### The ceftriaxone-induced intestinal dysbiosis in BALB/c mice

**Figure 2:**
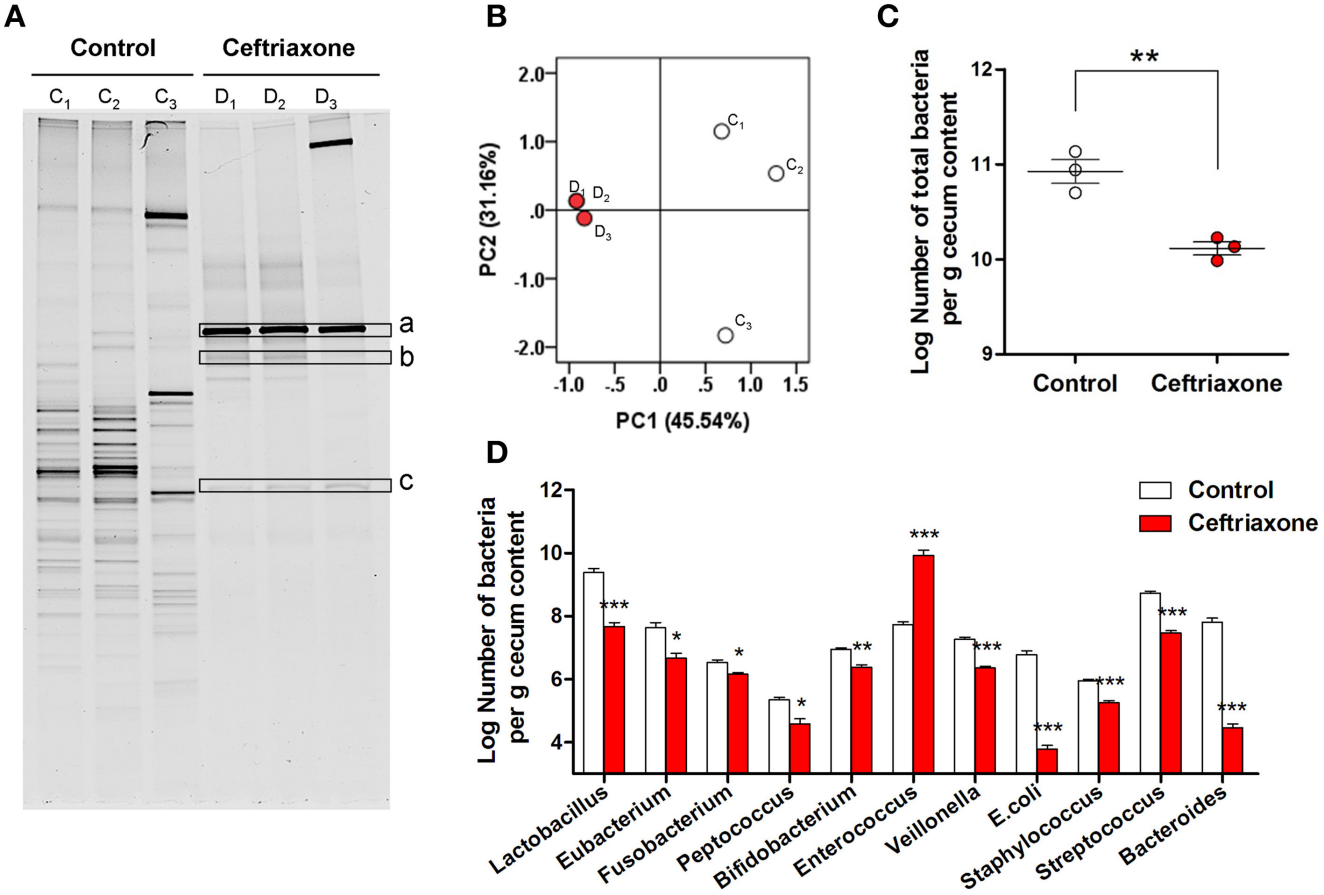
**Ceftriaxone induced intestinal dysbiosis in mice. (A)** DGGE pattern of the cecal microbial community of mice. C_1_–C_3_, control mice, D_1_–D_3_, dysbiosis mice. a–c, the bands differ from control were extracted from the DGGE gel and sequenced. **(B)** PCA of the DGGE profile. Community similarity was calculated using the weighted UniFrac distance metric, which incorporates phylogenetic as well as relative abundance information. PC1 and PC2 account for 76.70% of the variation. White dots, healthy mice; red dots, ceftriaxone-treated mice. **(C)** The total population of intestinal microbes in control and ceftriaxone-treated mice detected by qPCR. **(D)** The population of selected commensal bacteria in control and ceftriaxone-treated mice detected by qPCR. ^*^p < 0.05; ^**^p < 0.01; ^***^p < 0.001.

DGGE fingerprinting of cecal bacteria indicated a significant decrease of microbial diversity in ceftriaxone-treated mice. The number of bands was reduced dramatically from 27.004 ± 1.000 to 7.667 ± 0.333 (Figure 2A, *p* = 0.0001). The data resulting from DGGE analysis were subjected to PCA. Figure 2B shows that distinct intestinal microbiota profiles are present in the control mice and the ceftriaxone-treated mice. Samples taken at day 8, right after the 7-day's ceftriaxone administration, were typically of the same group (D_1_, D_2_, D_3_), in contrast to samples from the control mice (C_1_, C_2_, C_3_) which were treated sterile water. The Log number of total bacteria per gram of cecum content as assessed by q-PCR decreased from 10.942 ± 0.125 to 10.116 ± 0.070 (Figure 2C, *p* = 0.0049). Contrary to the decreasing pattern of overall bacterial population, several bands in the antibiotic-treated samples were more prominent (Figure 2A, a–c). Sequence analysis of 16S rRNA gene fragments of these bands revealed that a and b belonged to the genus *Enterococcus*, and c showed highest similarity to *Clostridium* species (Figure S2).

Population changes of 11 selected commensal bacterial community members were also assessed by qPCR. Compared with control mice, the number of *Escherichia coli*, *Streptococcus* spp., *Veillonella* spp., *Bacteroides-Prevotella-Porphyromonas*, and *Lactobacillus* groups in the cecal contents of antibiotic-treated mice decreased (Figure 2D, all *p* < 0.001). In contrast, because of tolerance to ceftriaxone, the Log number of *Enterococcus* spp. increased markedly from 7.732 ± 0.084 to 9.913 ± 0.178 (*p* = 0.0004), which is consistent with the DGGE results.

### Dysfunction of intestinal mucosal barrier in mice treated with ceftriaxone

**Figure 3:**
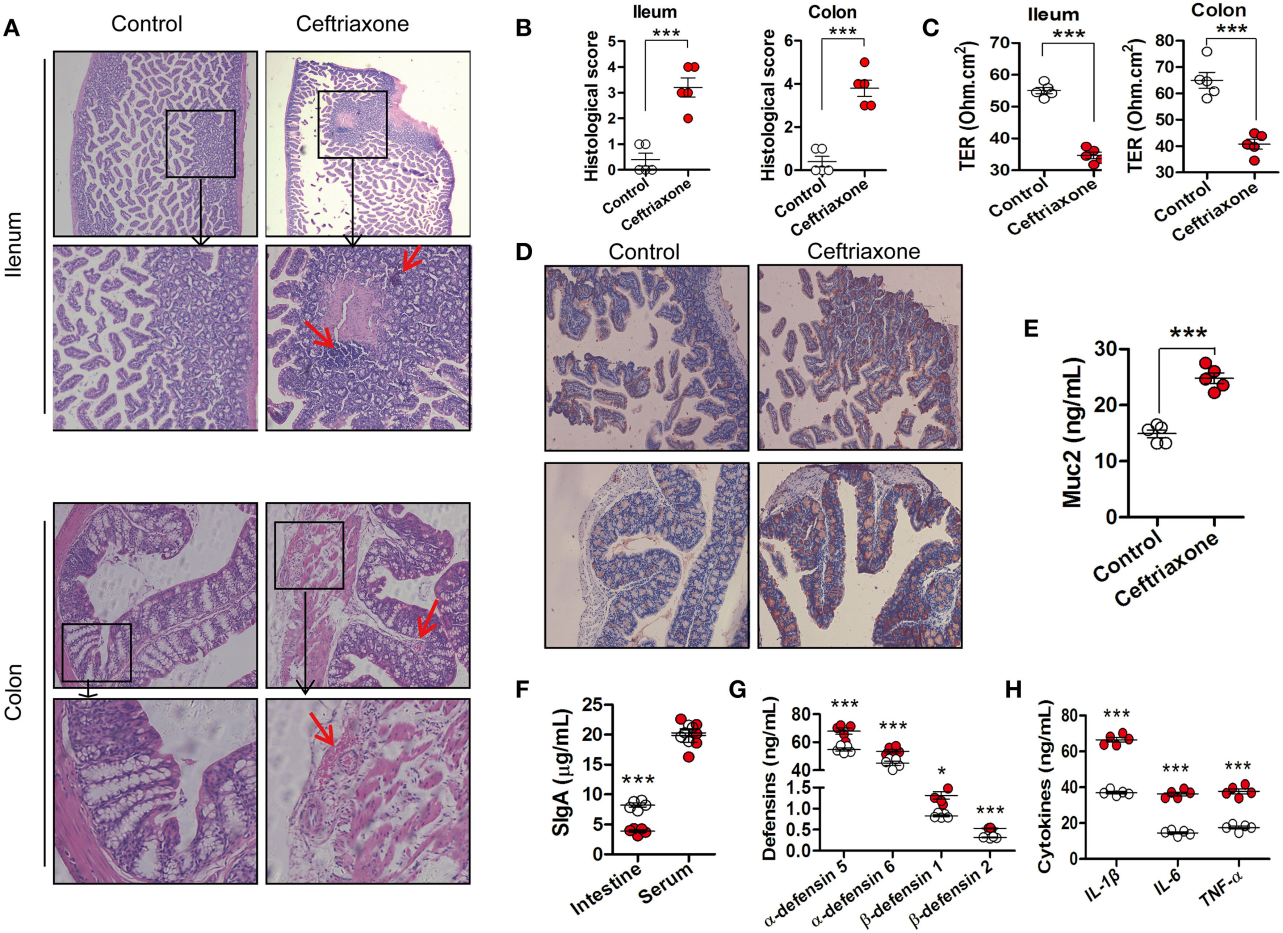
**The intestinal phenotype of ceftriaxone-treated mice. (A)** Representative patterns of HE-stained sections of distal ileum and colon in mice. Magnification, ×200. The red arrows indicate inflammatory cell infiltration, or vascular dilatation and congestion. **(B)** Histological evaluation of the HE-stained sections. **(C)** Trans-epithelial resistance (TER) of mouse distal ileum and colon was determined by measuring the average changes in potential difference in response to 3 μA current generated across the tissue segments every 6 s for 30 min. **(D)** Muc2 immunostaining in mouse distal ileum (top) and proximal colon (bottom), Magnification, ×200. **(E)** The concentration of Muc2 in intestinal mucus of mice. **(F)** The concentration of SIgA in intestinal mucus and serum IgA of mice. **(G)** The concentration of defensins in intestinal mucus of mice. **(H)** Serum levels of IL1-ß, IL-6, and TNF-α in mice. ^*^p < 0.05; ^***^p < 0.001. All the samples were taken at day 8.

After sectioning and HE staining, ileum and colon samples were observed under a microscope. Lesions and inflammatory cells infiltration were observed in the ileum of ceftriaxone-treated mice (Figure 3A, top). The integrity of microvilli in the ileum of ceftriaxone-treated mice was perturbed, they arranged irregularly, and in some instances were desquamated, and the local membrane of the intestinal mucosal epithelial cell is not complete (Figure S3). The ceftriaxone-treated mice had an increase in hyperplasia of the colonic mucosa and distorted tissue architecture; they had more severe vascular dilatation and congestion than that of control mice (Figure 3A, bottom). The histological score (Figure 3B) of both the ileum and colon in mice treated with ceftriaxone was higher compared with control mice.

The TER of the distal ileum and proximal colon in ceftriaxone-treated mice was reduced by 36.90 and 37.30% (Figure 3C, all *p* < 0.0001), respectively, compared with control mice, suggesting a dramatic increase of intestinal permeability.

Figure 3D shows representative images of distal ileum and proximal colon mucosae stained for Muc2 protein. The expression of Muc2 was significantly increased in ceftriaxone-treated mice compared with control mice (Figure 3D). Muc2 protein content of the intestinal mucus also increased by approximately 76.70% (*p* = 0.0001; Figure 3E). A significant decrease of SIgA expression (Figure 3F, *p* = 0.0001), and increases of α-defensin 5 (*p* = 0.0003), α-defensin 6 (*p* = 0.0007), β-defensin 1 (*p* = 0.0360), and β-defensin 2 (*p* = 0.0001) expression (Figure 3G) in the intestinal fluid of ceftriaxone-treated mice were observed. In contrast, serum IgA levels were not affected (*p* = 0.7269). The concentrations of inflammatory cytokines in serum of ceftriaxone-treated mice increased (Figure 3H, all *p* < 0.001).

### FMT and BCT promote re-establishment of the intestinal microecology

FMT and BCT were performed on ceftriaxone-treated mice for 3 consecutive days. To perform BCT, we cultured a diverse collection of 11 species from the fecal content of healthy mice using selective media, including representatives of 5 phyla that constitute the majority of the mammalian intestinal microbiota (*Firmicutes*, *Proteobacteria*, *Actinobacteria*, *Bacteroidetes*, and *Fusobacteria*; Table 1) (Mitsuoka, 1978). The proportion of each group was defined according to the fecal contents of control mice. Because of the highly elevated population of *Enterococcus* spp. in ceftriaxone-treated mice, this group was excluded from the bacterial consortium.

**Figure 4:**
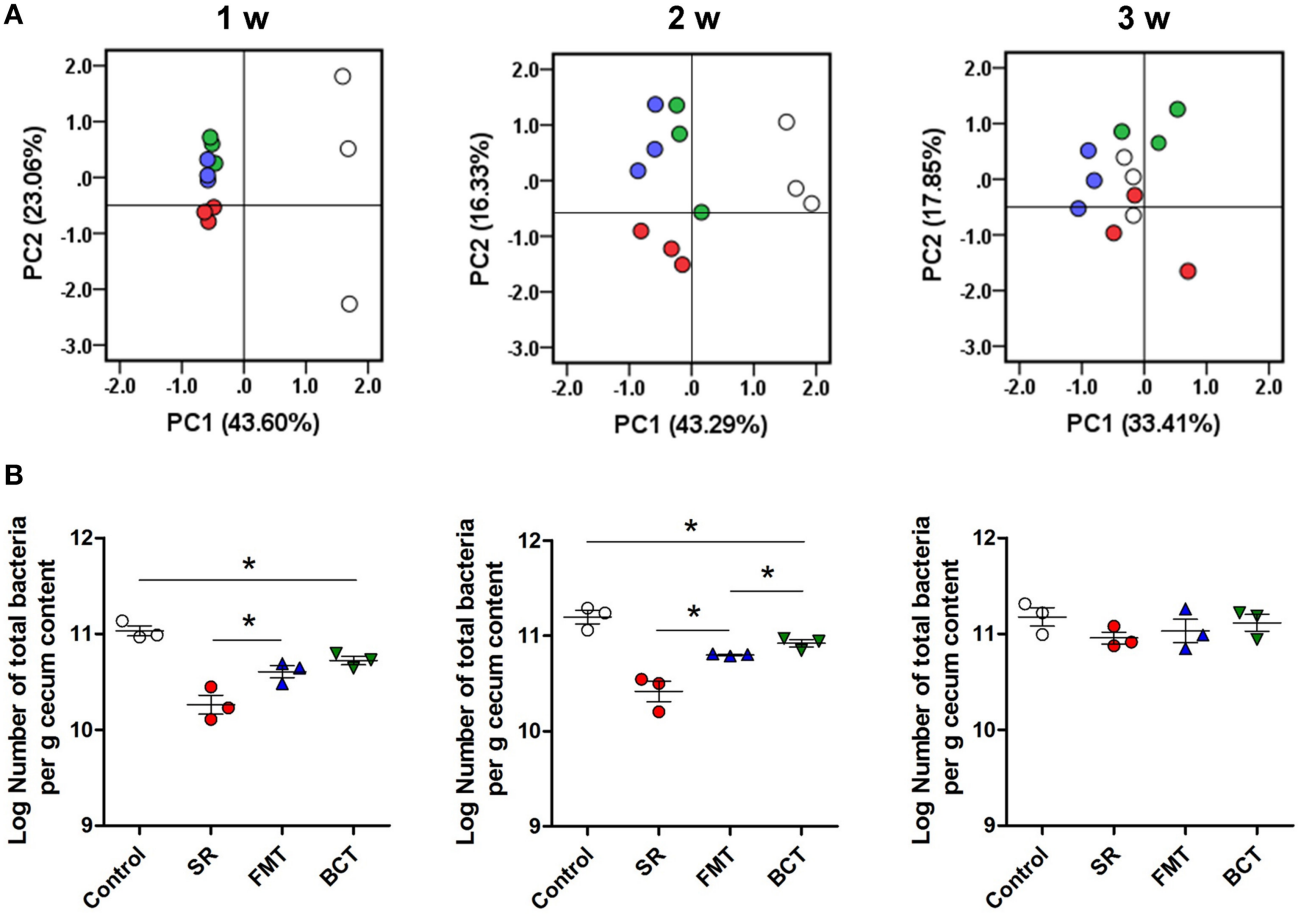
**The recovery of intestinal microbiota in different mice groups. (A)** PCA of the cecal microbiota in different experimental mice groups during the 3-week recovery. PC1 and PC2 account for 66.66, 59.62, and 51.26% of the variation in different weeks. Each symbol represents one microbiota (dot). White dots, healthy mice; red dots; SR mice, blue dots, FMT mice; green dots, BCT mice. **(B)** The population of total intestinal microbes in different mice groups detected by qPCR. ^*^p < 0.05; w, week(s) after ceftriaxone treatment.

The cecal microbiota in transplanted mice was analyzed by DGGE and qPCR, and the results are shown in Figure 4. By the first week (4 days after transplantation), the diversity and population (total numbers) of the microbiota in the FMT and BCT groups was higher than in the SR group, but was still different from the control mice. There was a significant difference in the total number of bacteria between the FMT/BCT groups and the SR group (*p* = 0.0427 and *p* = 0.0131, respectively). The abundance of total bacteria in the BCT and FMT groups was much lower in both of these groups than in the control group (*p* = 0.0322). After 2 weeks of recovery, increasing patterns of microbial diversity were observed in the SR, FMT, and BCT groups, although the total bacterial populations were still lower than the control group (*p* = 0.0037, *p* = 0.0050, and *p* = 0.0265, respectively). Compared with the SR group, the total number of microbes in the FMT/BCT group was larger (*p* = 0.0230; *p* = 0.0112). A significant difference was detected between the total number of microbes in the BCT and FMT groups (*p* = 0.0355). After 3 weeks of recovery, both the diversity and population of different groups were similar to those of the control group, suggesting that total recovery of the intestinal microbial structure had occurred.

Population changes of the 11 selected bacterial groups were also detected during recovery (Table 2). By the first week, the numbers of bacterial groups that had recovered (defined as having no significant difference compared with control) in the cecum of mice from the SR, FMT, and BCT were two, three, and five, respectively. After 2 weeks, these numbers had increased to five, six, and seven. By the third week, all of the selected groups in the FMT and BCT groups had recovered to the level of control mice, but the bacterial populations in the SR group were variable; the populations of *Eubacterium rectale-Clostricium coccoides* group and *E. coli* were less than the control, while in contrast, the *Enterococcus* population was still larger.

**Table 2:** **Quantification of selected commensal bacteria in different mice groups during recovery**.

	**Control**			**SR**			**FMT**			**BCT**		
	**1 week**	**2 weeks**	**3 weeks**	**1 week**	**2 weeks**	**3 weeks**	**1 week**	**2 weeks**	**3 weeks**	**1 week**	**2 weeks**	**3 weeks**
*Lactobacillus* group	9.383 ± 0.203	9.385 ± 0.203	9.382 ± 0.203	8.173 ± 0.237	8.677 ± 0.204	9.479 ± 0.286^*^	8.628 ± 0.143	9.278 ± 0.189^#^	9.357 ± 0.190^#^	8.723 ± 0.151	9.221 ± 0.250^∇^	9.524 ± 0.104^∇^
*Eubacterium rectale-Clostridium coccoides* group	7.575 ± 0.229	7.624 ± 0.279	7.558 ± 0.226	6.700 ± 0.120	6.896 ± 0.075	7.086 ± 0.056	6.824 ± 0.047	7.349 ± 0.193	7.536 ± 0.302^#^	7.383 ± 0.195^∇^	7.328 ± 0.105	7.648 ± 0.204^∇^
*Fusobacterium* spp.	6.609 ± 0.150	6.532 ± 0.142	6.534 ± 0.142	6.498 ± 0.143^*^	6.630 ± 0.121^*^	6.594 ± 0.067^*^	6.443 ± 0.085^#^	6.607 ± 0.091^#^	6.672 ± 0.125^#^	6.515 ± 0.063^∇^	6.559 ± 0.056^∇^	6.588 ± 0.081^∇^
*Peptococcus* spp.	5.351 ± 0.126	5.382 ± 0.091	5.351 ± 0.126	5.142 ± 0.137^*^	5.356 ± 0.068^*^	5.476 ± 0.091^*^	5.141 ± 0.153^#^	5.270 ± 0.073^#^	5.311 ± 0.028^#^	5.255 ± 0.217^∇^	5.359 ± 0.078^∇^	5.501 ± 0.016^∇^
*Bifidobacterium* spp.	6.967 ± 0.080	6.911 ± 0.154	6.937 ± 0.084	6.444 ± 0.078	6.743 ± 0.074	6.832 ± 0.050^*^	6.577 ± 0.091	6.647 ± 0.044	7.031 ± 0.290^#^	6.671 ± 0.109	6.750 ± 0.092	6.906 ± 0.252^∇^
*Enterococcus* spp.	7.732 ± 0.146	7.549 ± 0.119	7.550 ± 0.119	9.346 ± 0.208	9.030 ± 0.094	8.131 ± 0.094	9.198 ± 0.129	8.573 ± 0.228	7.573 ± 0.228^#^	9.224 ± 0.225	8.531 ± 0.270	7.531 ± 0.270^∇^
*Veillonella* spp.	7.265 ± 0.116	7.275 ± 0.881	7.344 ± 0.069	7.026 ± 0.043	7.289 ± 0.056^*^	7.330 ± 0.108^*^	7.135 ± 0.111^#^	7.671 ± 0.458^#^	7.615 ± 0.084	7.295 ± 0.144^∇^	7.364 ± 0.096^∇^	7.358 ± 0.076^∇^
*E. coli*	6.776 ± 0.226	6.881 ± 0.172	6.808 ± 0.169	4.792 ± 0.190	6.121 ± 0.022	6.420 ± 0.203	5.706 ± 0.212	6.932 ± 0.059^#^	6.950 ± 0.093^#^	5.349 ± 0.101	6.788 ± 0.143^∇^	6.846 ± 0.403^∇^
*Staphylococcus* spp.	5.950 ± 0.065	5.964 ± 0.041	6.093 ± 0.099	5.563 ± 0.131	6.049 ± 0.128^*^	6.164 ± 0.105^*^	5.536 ± 0.140	5.823 ± 0.027	5.997 ± 0.085^#^	5.976 ± 1.134	6.097 ± 0.119^∇^	6.097 ± 0.124^∇^
*Streptococcus* spp.	8.795 ± 0.121	8.566 ± 0.098	8.527 ± 0.225	7.536 ± 0.217	7.702 ± 0.533	8.696 ± 0.206^*^	7.772 ± 0.169	8.242 ± 0.307	8.536 ± 0.186^#^	7.939 ± 0.050^∇^	8.293 ± 0.248	8.367 ± 0.062^∇^
*Bacteroides-Prevotella-Porphyromonas*	8.075 ± 0.126	8.225 ± 0.511	8.031 ± 0.400	6.137 ± 0.064	8.304 ± 0.306^*^	8.220 ± 0.271^*^	6.211 ± 0.151	8.290 ± 0.373^#^	8.145 ± 0.279^#^	7.047 ± 0.091	8.384 ± 0.234^∇^	8.031 ± 0.512^∇^

*The population of selected commensal bacteria per 1 g of mice cecal contents was detected by qPCR. Means ± SD of 5 mice per group are shown*.

* *The population in SR mice is similar to control*;

# *The population in FMT mice is similar to control*;

∇ *The population in BCT mice is similar to control. w, week(s) after ceftriaxone treatment*.

### Effects of FMT and BCT on the repair of mechanical barriers

**Figure 5:**
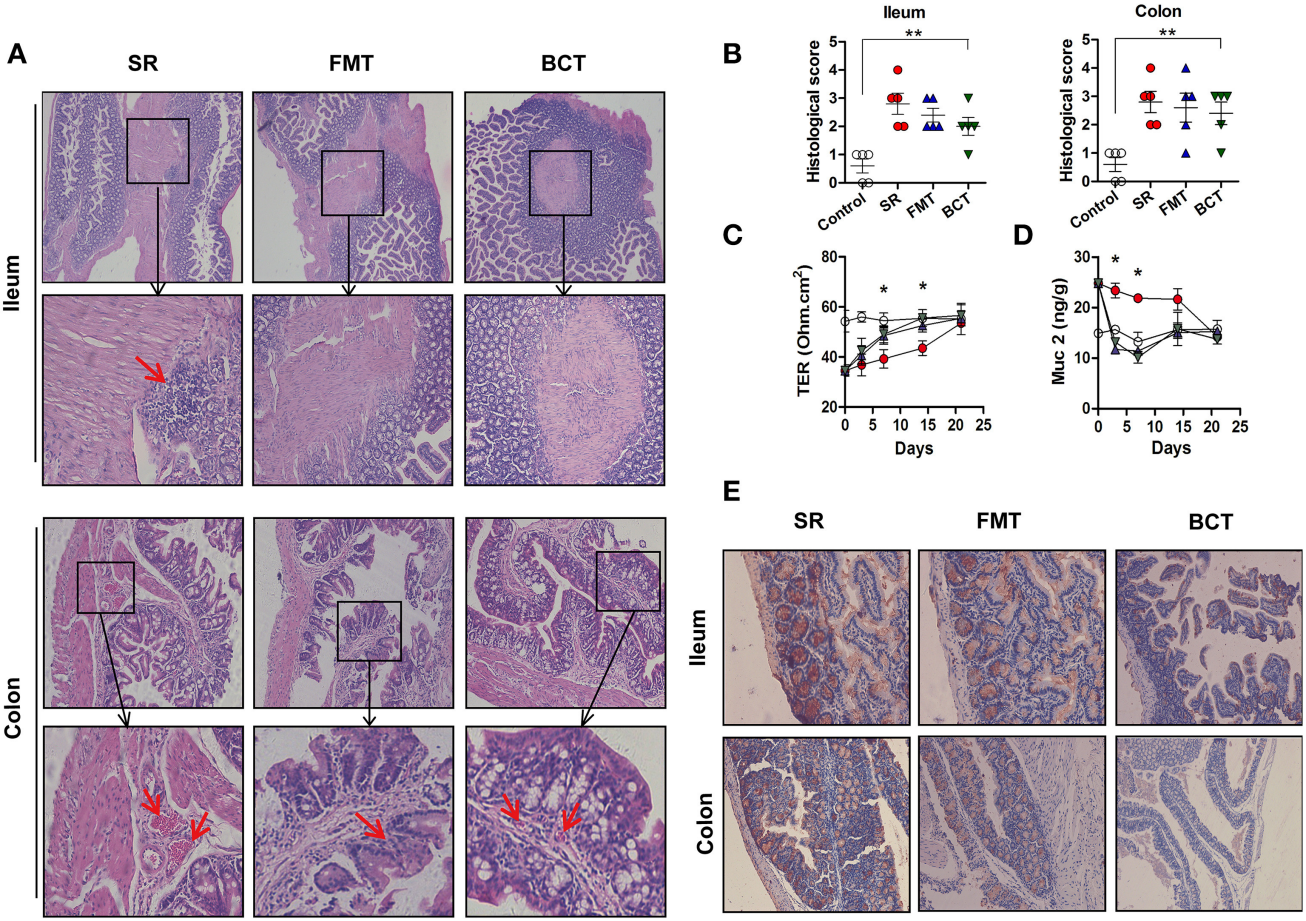
**Post-FMT or BCT changes of the mechanical barriers in intestinal mucosa of different mice groups. (A)** Representative patterns of HE-stained sections of distal ileum and proximal colon in mice after 1 week recovery. Magnification, ×200. The red arrows indicate inflammatory cell infiltration, or vascular dilatation and congestion. **(B)** Histological analysis was performed after 1 week recovery. ^**^p < 0.001 compared with control. **(C)** TER of distal ileum in different mice groups detected after 1 week recovery, ^*^, FMT compared with SR, *p* < 0.05. The X axes indicate days after ceftriaxone treatment. **(D)** The concentration of Muc2 in intestinal mucus of mice. ^*^, FMT compared with SR, *p* < 0.05. All values are means ± SD of 5 mice per group. **(E)** Muc2 immunostaining in mouse distal ileum and proximal colon.

Histological examination after 1 week of recovery showed that there were still lesions and inflammatory cells infiltration in distal ileum of the antibiotic treated mice (Figure 5A, top), distorted tissue architecture and vascular congestion were also detected (Figure 5A, bottom). While the destruction of the mucosae appeared to have been substantially ameliorated in mice receiving FMT or BCT, as less inflammatory cells infiltration, and less distorted tissue architecture and vascular congestion were detected, but these were not statistically significant according to the histological evaluation (Figure 5B, SR vs. FMT/BCT, all *p* > 0.05). After 2 weeks, the mucosae in the three different treatment groups had recovered to the level of the control group (Figures S4, S5). The intestinal mucosal permeability of mice decreased gradually during the recovery period (Figure 5C). Compared with the SR group, the mucosal permeability of mice in the FMT and BCT groups was significantly lower after 1 (*p* = 0.0483, *p* = 0.0465) and 2 (*p* = 0.0384, *p* = 0.0228) weeks of recovery.

Along with the increase in microbial density, the expression level of Muc2 decreased gradually, and compared with the SR group, Muc2 expression in the FMT and BCT groups was much lower (Figure 5E). This is consistent with the ELISA results for intestinal mucus in these mice (Figure 5D). Muc2 secretion in the FMT and BCT groups on the 3rd and 7th day of recovery had reached a level close to that observed in the control group, which was significantly lower than that in the SR group (*p* = 0.0385, *p* = 0.0221), but there was no significant difference observed between the two transplanted groups.

### Effects of FMT and BCT on the immune barrier

**Figure 6:**
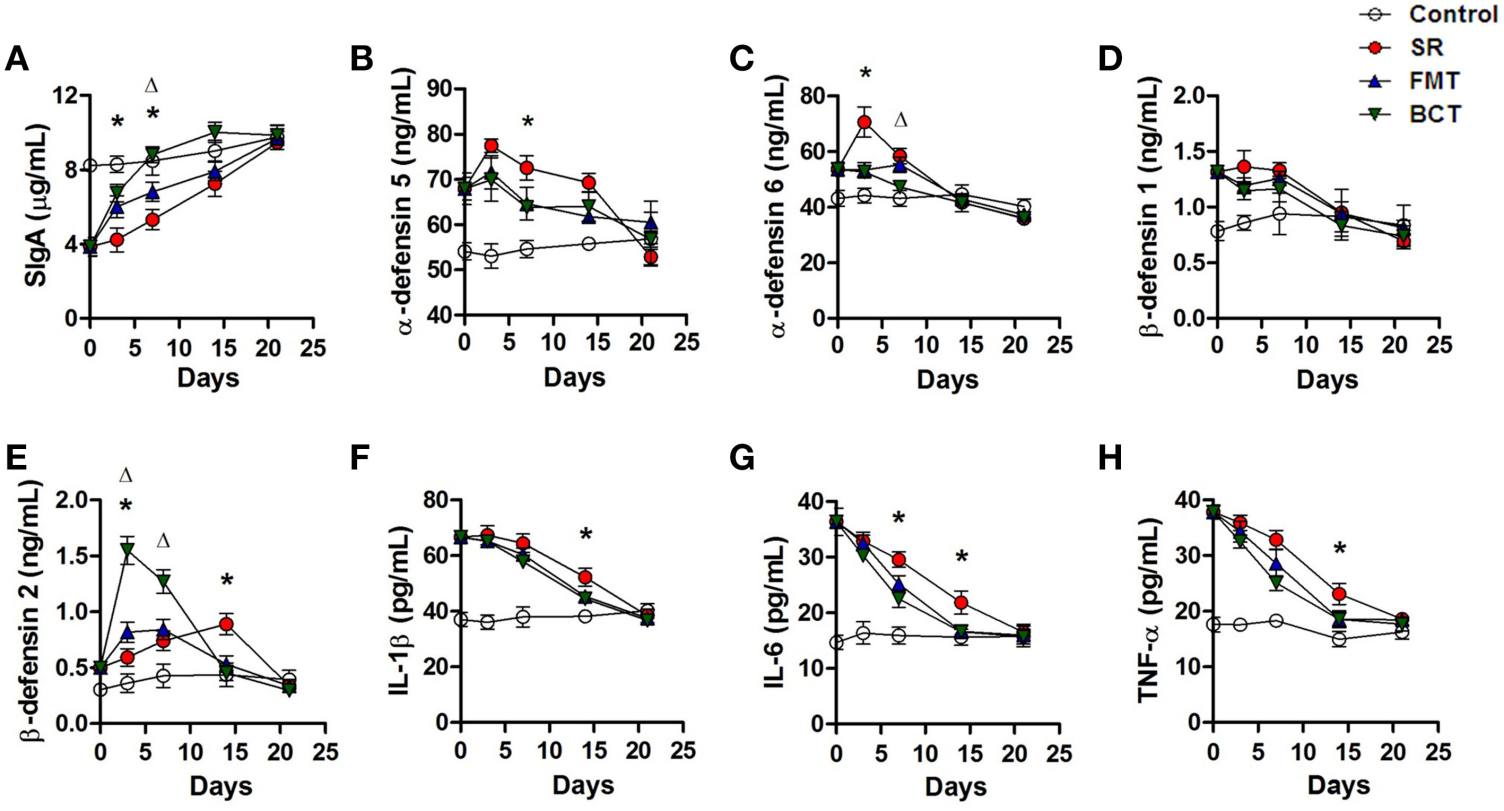
**Post-FMT or BCT changes of intestinal SIgA, defensins and serum inflammatory cytokines**. The concentration of SIgA **(A)**, α-defensin 5 **(B)**, 6 **(C)**, β-defensin 1 **(D)**, and 2 **(E)** in intestinal mucus of mice, and the serum levels of IL1-β **(F)**, IL-6 **(G)**, TNF-α **(H)** in mice post-FMT or BCT were measured by ELISA. Values are means ± SD of 5 mice per group. ^*^, FMT compared with SR *p* < 0.05. ^Δ^, BCT compared with FMT *p* < 0.05. The X axes indicate days after ceftriaxone treatment.

An increasing SIgA concentration was observed in the intestinal mucus of mice during recovery (Figure 6A). Among the different experimental groups, the BCT group showed a clearly superior recovery. On the 3rd and 7th day, the concentration of SIgA in the BCT group was significantly higher than in the SR group (*p* = 0.0230, *p* = 0.0340), and was higher than the FMT group by the 7th day (*p* = 0.0320). The concentration of SIgA in the FMT group recovered faster than the SR group, although the differences on the 3rd and 14th day were not significant statistically.

Higher concentrations of intestinal defensins (α-defensins 5 and 6, β-defensins 1 and 2) were detected in mice treated with ceftriaxone (Figure 3G). During recovery, the secretion of these defensins in the SR group did not decrease, but instead continued to increase for at least 3 days, then decreased gradually until the third week after treatment when levels reached those of controls (Figures 6B–E). Transplantation of fecal microbiota or bacterial consortium promoted reduction of α-defensins 5 and 6; on day 7, intestinal levels of α-defensin 5 in the FMT and BCT groups were much lower than in the SR group (*p* = 0.0479, *p* = 0.0477). The level of α-defensin 6 in the FMT and BCT groups started to decrease immediately after transplantation, and was significantly lower than the SR group by the 3rd day (*p* = 0.0281, *p* = 0.0273). By day 7, the level of α-defensin 6 in the BCT group was lower than that in the FMT group (*p* = 0.0394). The levels of β-defensin 1 in the FMT and BCT groups decreased gradually after transplantation, but no significant differences were detected compared with the SR group (all *p* > 0.05). The concentration of β-defensin 2 in the FMT and BCT groups increased dramatically during transplantation to levels that were significantly higher than those in the SR group (*p* = 0.0130, *p* = 0.0460) and in the control group (*p* = 0.0080, *p* = 0.0010), and this was followed by a sharp decreasing. By the second week, β-defensin 2 levels in the FMT and BCT groups had declined to the level seen in the control group, which was significantly lower than that in the SR group (*p* = 0.0351, *p* = 0.0243).

During recovery, the concentration of serum IL-1β in all mice gradually declined (Figure 6F). In the first week, there were no significant differences observed between the three groups (all *p* > 0.05). By the second week, the reduction in serum IL-1β levels in the microbiota-transplanted mice was more obvious than in SR mice (SR vs. FMT, *p* = 0.0484; SR vs. BCT, *p* = 0.0484); however, no significant difference was observed between the FMT and BCT groups (*p* > 0.05). The deceasing pattern of serum IL-6 and TNF-α levels observed during recovery was similar to that of IL-1β (Figures 6G,H). After 3 weeks, the serum levels of inflammatory cytokines in all the groups had reduced to the normal level (i.e., *p* > 0.05 compared with the control group).

## Discussion

As shown in many studies (Wlodarska et al., 2011; Aguilera et al., 2015; Grasa et al., 2015), treatment with antibiotics has significant effects on the structure of the intestinal microbiota. Our study also demonstrated that intragastric ceftriaxone induced severe dysbiosis in mice, and both the population and diversity of microbes were disturbed in the cecum of antibiotic-treated mice. The cecum is connected to the ileum and is considered to be the beginning of the large intestine. There are large numbers of bacteria dwelling in this cavity, therefore, we analyzed the bacterial contents in the cecum to investigate the effects of microbiota transplantation in the small and large intestines. Consequently, the barrier function of the distal ileum and colon was evaluated. The spontaneous recovery of intestinal microbiota in mice after antibiotic treatment takes at least 3 weeks to become restored to the previous level (Lawley et al., 2009), and in many cases, the recovery is unpredictable and incomplete (Dethlefsen and Relman, 2011). We observed that, during the first week of recovery, compared with SR mice, the population and diversity of intestinal microbiota in the cecum in the FMT and BCT groups were larger. By the end of the second week, there was a significant difference in total bacterial abundance between the FMT and BCT groups, suggesting that BCT can accelerate the increase of microbial diversity and population in the mouse intestine.

Unexpectedly, histological lesions were detected in the small intestine of mice treated with ceftriaxone, and vascular dilation and congestion were observed in the large intestine. These observations collectively suggest a proinflammatory state of the mouse intestine. The disrupted organization of microvilli and increased permeability of the intestinal mucosa suggest a deficiency in nutrient absorption (Bennett et al., 2014), and risk of bacterial endotoxin translocation (Strowski and Wiedenmann, 2009). These findings were confirmed by the elevation of circulatory proinflammatory cytokines, which suggested that the local inflammatory responses in the intestine affected the changes in systemic immunity. Antibiotics are often used to create germ-free conditions; however, the intestinal balance of microbiota is disrupted, leading to greater susceptibility to infection in the intestine. Our result seems to differ from the traditional view; however, it also suggested the importance of protecting commensal bacteria. As we can see from the data, compared with SR, FMT and BCT ameliorated the increased permeability of the intestinal mucosa, which indicates an important role of the microbiota in maintaining the normal intestinal epithelial structure.

The intestinal mucosal surfaces are coated with a layer of viscous mucus that protects the epithelial cells from chemical, enzymatic, microbial, and mechanical insult. Muc2 is the major component of the mucus layer in the small and large intestines. Increased intestinal mucus production in mice with dysbiosis may be one of the causes of antibiotic-associated diarrhea because hypersecretion of glycoproteins by the intestinal mucosa is observed during acute infection (Hasnain et al., 2011). It is reported that Muc2 helps the disassociation of pathogenic and normal microbiota from the intestinal mucosa, and thus prevents infectious colitis (Bergstrom et al., 2010), and Muc2-deficient mice develop spontaneous colitis (Wenzel et al., 2014). We therefore infer that increased Muc2 is a compensatory secretion from the mucosal goblet cells in the absence of the microbial barrier. Another possible reason is that many inflammatory factors such as IL-1β, IL-6, interferons, and TNF-α can quickly upregulate expression of mucins (Linden et al., 2008), and that ceftriaxone-induced dysbiosis may have caused the observed increase in inflammatory cytokines, thus stimulating the secretion of Muc2. During the 3 days of FMT and BCT, the expression and secretion of Muc2 decreased rapidly and remained stable until the end of the experiment. On the one hand, metabolites of the intestinal microbiota can stimulate expression of mucins by goblet cells (Lutgendorff et al., 2008). On the other hand, they can also rapidly metabolize excessive mucins (Macfarlane et al., 2005); therefore, the decreased bacterial population may also contribute to the increased amount of Muc2 in the intestinal mucus. This illustrates that the intestinal microbiota play crucial roles in regulating the equilibrium of intestinal mucus.

Intestinal mucus provides a large matrix for a rich array of antimicrobial molecules such as SIgA and defensins. They are essential components of the innate immune system and contribute greatly to intestinal barrier function. SIgA is the most abundant immunoglobulin found in intestinal mucus (Corthesy, 2013). Our results showed that SIgA secretion in ceftriaxone-treated mice was significantly lower than in control mice, suggesting a significant reduction in the protective ability of the intestinal mucosa against exogenous bacterial infection. SIgA molecules are assembled from proteins expressed in two cell lineages. The heavy and light chains and the J chain are produced in plasma cells, whereas the secretory component (SC) is associated with the immunoglobulin complex during transcytosis across the epithelial layer (Corthesy and Spertini, 1999). When measuring serum levels of IgA, we detected no significant difference between the ceftriaxone-treated mice and healthy controls (Figure 3E). This suggests that ceftriaxone-induced dysbiosis may have affected the expression of SC at the intestinal epithelial layer, resulting in reduced SIgA levels in the intestine. Compared with SR, the FMT and BCT methods restored the secretion of SIgA more effectively, and there was also a significant difference between FMT and BCT.

Defensins are endogenous antibiotics with microbicidal activity against a wide range of microbes. They provide a critical mucosal defense and can signal to various components of the innate and adaptive immune systems (Wehkamp et al., 2005). When the intestinal ecosystem is in balance, epithelial cells produce defensins such as α-defensins 5 and 6, and β-defensin 1, as a strong chemomechanical barrier to control the intestinal microbiota and maintain immunological homeostasis. Upregulation of α-defensin 5 is a predictor of chronic/relapsing pouchitis, and can mediate shifts in the composition of the microbiota that favor inflammation (Scarpa et al., 2012). During inflammation or infection, additional defensins such as β-defensin 2 and 3 are induced as a type of “demand system” (Semple and Dorin, 2012). Here, we observed that the levels of α-defensins 5 and 6, and of β-defensins 1 and 2, in the intestinal mucus of ceftriaxone-treated mice were significantly higher than those of control mice, which suggests that dysbiosis of the intestinal microbiota had caused a susceptible state in the mouse intestine. These increased small peptides may further exert their antimicrobial activity toward intestinal microbiota and cause more severe dysbiosis. As shown in Figure 6, in SR mice, these defensins continuously increased for at least 1 week after antibiotic administration was stopped. In contrast, levels of α-defensins 5 and 6, and β-defensin 1, decreased gradually after FMT or BCT, and marked differences were detected between the SR group and the FMT/BCT groups by the first week.

The secretion of β-defensin 2 in mouse intestine during FMT/BCT was affected in a *negative feedback pattern*. It increased rapidly, reaching the highest level at day 3, and this was followed by a sharp decline to the control level within 2 weeks. A systemic immune response has previously been described in patients treated with FMT, including fever and a temporary increase of C-reactive protein (Vermeire et al., 2012; Angelberger et al., 2013). Our results suggest that transplantation of microbes may aggravate the inflammatory responses in mice with intestinal dysbiosis, and the impact of the BCT method seems stronger. Nevertheless, compared with FMT and BCT, the natural recovery of β-defensin 2 in ceftriaxone-treated mice took longer, suggesting a longer state of susceptibility in the mouse intestinal mucosa.

Proinflammatory cytokines such as IL-1β, IL-6, and TNF-α are well recognized as mediators during the onset of disease, and they play important roles in the local inflammatory reaction and immune response (Lim et al., 2014). As we showed, the levels of these cytokines were elevated after ceftriaxone treatment, which suggested that ceftriaxone-induced dysbiosis altered the systemic immune networks in these mice. These changes may correlate with a reduction in commensal bacteria and overgrowth of opportunistic pathogens. With the re-establishment of the microbial structure, levels of IL-1 β, IL-6, and TNF-α reduced gradually. Compared with the SR group, the FMT and BCT treatments promoted the reduction of proinflammatory cytokines, but no significant difference was found between these two groups. In addition, studies have shown that the stimulation of proinflammatory cytokines is closely associated with the expression of mucin (Ahn et al., 2005) and defensins (Krisanaprakornkit et al., 2000) by the intestinal mucosa, as well as an increase in mucosal permeability (Al-Sadi et al., 2008). The observed up- and down-regulation of cytokine levels are consistent with the changes of Muc2 and defensin levels during ceftriaxone treatment and over the course of recovery.

Overall, based on a short-term study, we have systematically examined changes in the intestinal mucosal phenotype, permeability, mucin expression, and expression of infection-related immune factors in BABL/c mice before and after transplantation with either fecal microbiota or a consortium of isolated commensal bacteria from healthy mice. Although limited by the low number of mice used at each collecting time point, our data still show that disruption of intestinal microbial homeostasis affects mucosal permeability, mucin expression, and the network of immune molecules. The FMT and BCT methods promote re-establishment of the intestinal microbial structure, and contribute to the temporal and spatial interplay between microbiota and intestinal mucosal barriers. The effects of the BCT method were comparable to those of FMT, and especially efficient in normalizing the secretion levels of SIgA and defensins. As a result of significant inter-individual variation and the possible risk of disease transmission, FMT is not regarded as a safe therapy, and there are also some aesthetic and ethical challenges associated with its use. In contrast, the bacteria used for BCT are purified from healthy donors, and the proportion of each bacterium can be standardized or individualized according to the different level of dysbiosis. BCT is therefore a viable alternative to conventional FMT for various gastrointestinal diseases. However, our results also suggest that transplantation of bacterial consortia may temporarily aggravate the inflammatory responses in recipients, and further detailed long-term studies should be conducted to evaluate these effects.

## Author contributions

LT designed the study; ML, PL, ZL, YW, GZ, HG, and SW were involved in experiment conduction and analysis; ML and PL performed data interpretation and wrote the manuscript; ML and PL contributed equally to this work.

### Conflict of interest statement

The authors declare that the research was conducted in the absence of any commercial or financial relationships that could be construed as a potential conflict of interest.

## Supplementary material

Figure S1 **The scheme of animal experiment**.

Figure S2 **The sequences of specific DGGE bands**.

Figure S3 **The intestinal phenotype of ceftriaxone-treated mice. (A)** Representative patterns of HE-stained sections of distal ileum in mice. Magnification, ×200. **(B)** Microvilli of ileum in healthy mouse and ceftriaxone-treated mouse. To observe the microvilli, the ultrathin sections were stained with uranyl acetate and lead citrate, and observed under a JEM-1400 TEM (Olympus, Japan). **(C)** Representative patterns of HE-stained sections of distal ileum in mice. Magnification, ×200. The red arrows indicate inflammatory cell infiltration, edema, or vascular dilatation and congestion.

Figure S4 **Post-FMT or BCT changes of the ileum in mice**. The pictures are representative patterns of HE-stained sections of distal ileum in mice after 1 and 2 week(s) recovery. Magnification, ×200. The red arrows indicate inflammatory cell infiltration, edema, or vascular dilatation and congestion.

Figure S5 **Post-FMT or BCT changes of the colon in mice**. The pictures are representative patterns of HE-stained sections of proximal colon in mice after 1 and 2 week(s) recovery. Magnification, ×200. The red arrows indicate inflammatory cell infiltration, edema, or vascular dilatation and congestion. The components of selective media are as follows: TATAC, Peptone 1.5 g, Yeast extraction 1 g, Sucrose 0.1 g, Tryptone 1 g, Esculin sesquihydrate 0.1 g, Agar 1.6 g, bovine serum 5 mL, TATAC I 2.2 ml, TATAC II 2.0 ml. TATAC I :Sodium azide 0.45 g, Sodium glutamate 30 g; TATAC II : Acridine orange 0.01 g, Triphenyl tetrazolium chloride (TTC) 0.1 g, Crystal violet (0.65%) 1.0 mL, Thallium sulfate 1.65 g. CS: EG Medium 100 mL, CS additive solution 5 mL, Goat blood 5 mL; CS additive solution: Sodium propionate 30 g, Neomycin sulfate 0.4 g, Colistin 0.02 g, Streptomycin sulfate 0.1 g. VS: Tryptone 0.5 g, Yeast extraction 0.3 g, Sulfur glycolic acid sodium 0.075 g, Ethyl Green (0.1%) 0.1 mL, Tween-80 0.1 mL, 50%Sodium lactate 2.5 mL, Agar 2 g, Goat blood 5 mL. PMS: EG Medium 100 mL, PMS additive solution 1 mL, PMS additive solution: Crystal violet 70 mg, Colorless slime mold toxin 50 mg. EC: Tryptone 2 g, Yeast extraction 0.05 g, Glucose 0.2 g, Na_2_HPO_4_ 0.04 g, KH_2_PO_4_ 0.04 g, Agar 2 g, NaN3 40 mg, TTC 10 mg. FS: EG Medium 100 mL, FS additive solution 1 mL; FS additive solution: Crystal violet 70 mg, Vacocin vancomycin 50 mg, Neomycin sulfate 300 mgBds: Peptone 2 g, Beef extract 1 g, Glucose 0.5 g, L-cysteine hydrochloride 0.05 g, Na_2_HPO_4_ 0. 4 g, Agar 2 g, Neomycin 0.01 g, Goat blood 5 mLThe media Eosin Methylene Blue, Baird-Parker, and Bifidobacterium BS Medium were purchased from Dalian United Stars Biology Tech. Co. Ltd.
