# Supplementary figures and images for: Fecal microbiota transplantation and bacterial consortium transplantation have comparable effects on the re-establishment of mucosal barrier function in mice with intestinal dysbiosis
Source: Front Microbiol. 2015 Jul 7;6:692. doi: 10.3389/fmicb.2015.00692 (PMC4493656; doi:10.3389/fmicb.2015.00692)

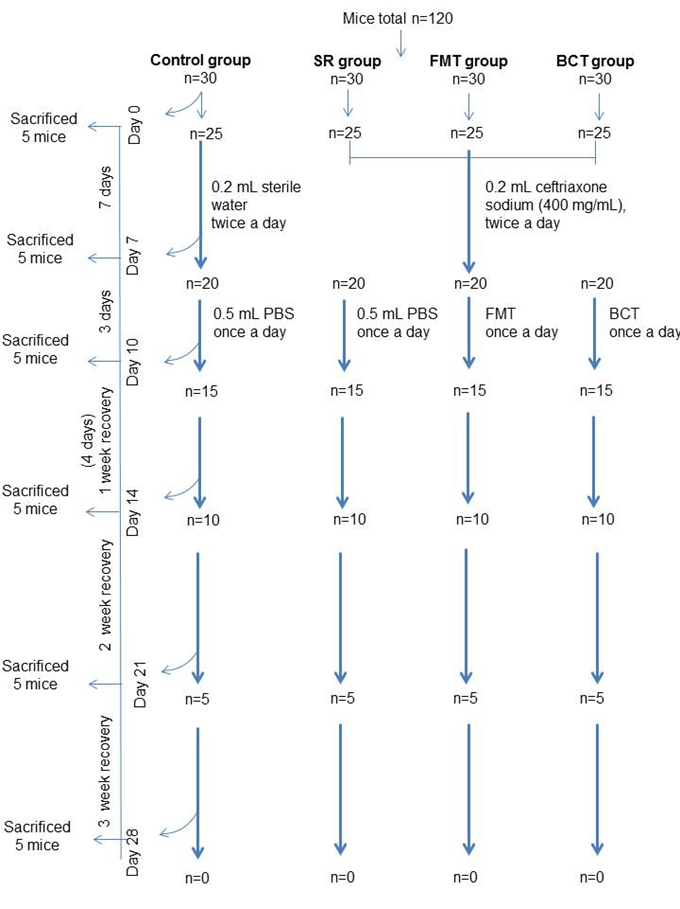

Supplement: Figure S1 — The scheme of animal experiment. [file Image1.TIF]

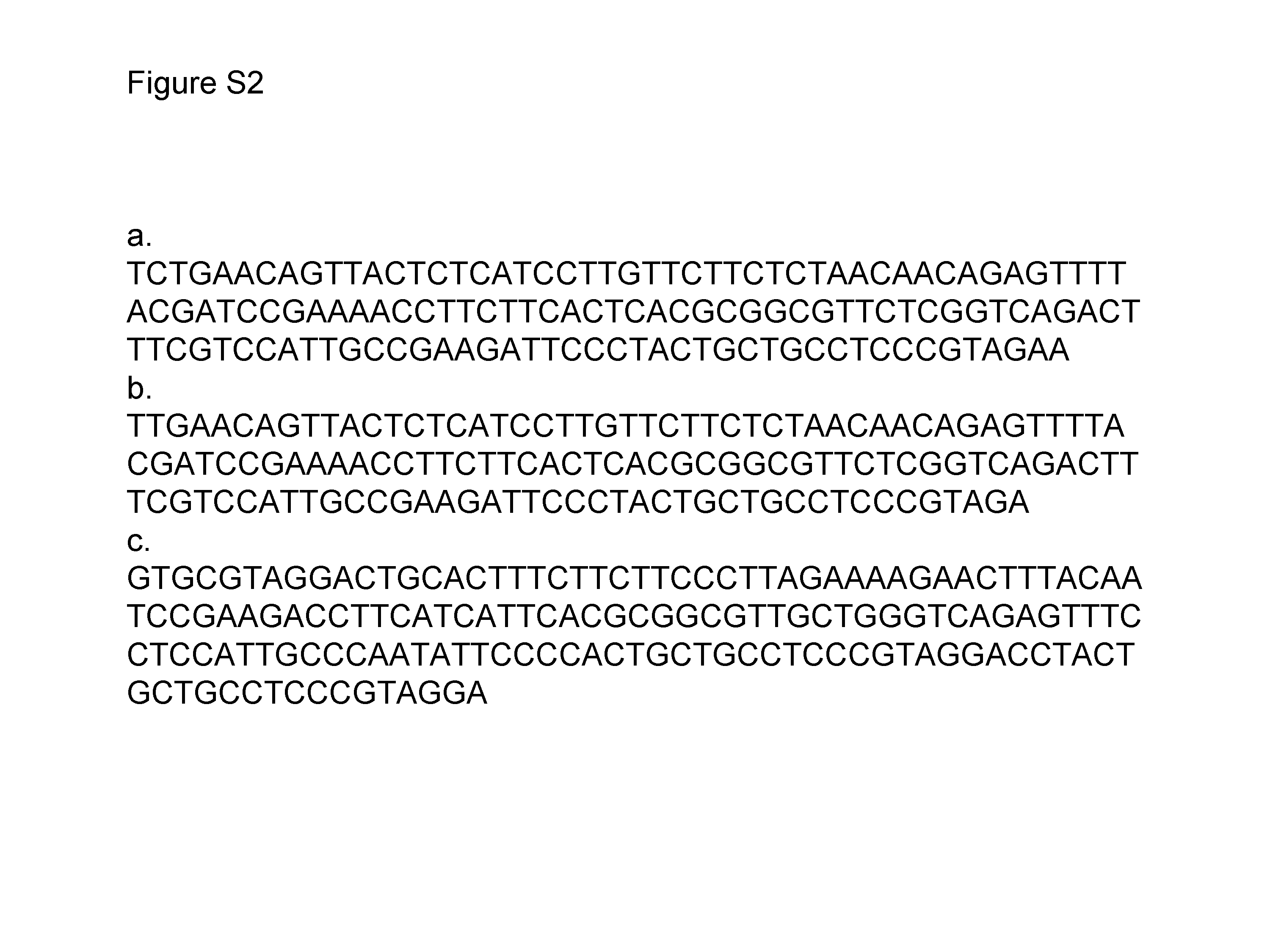

Supplement: Figure S2 — The sequences of specific DGGE bands. [file Image2.TIF]

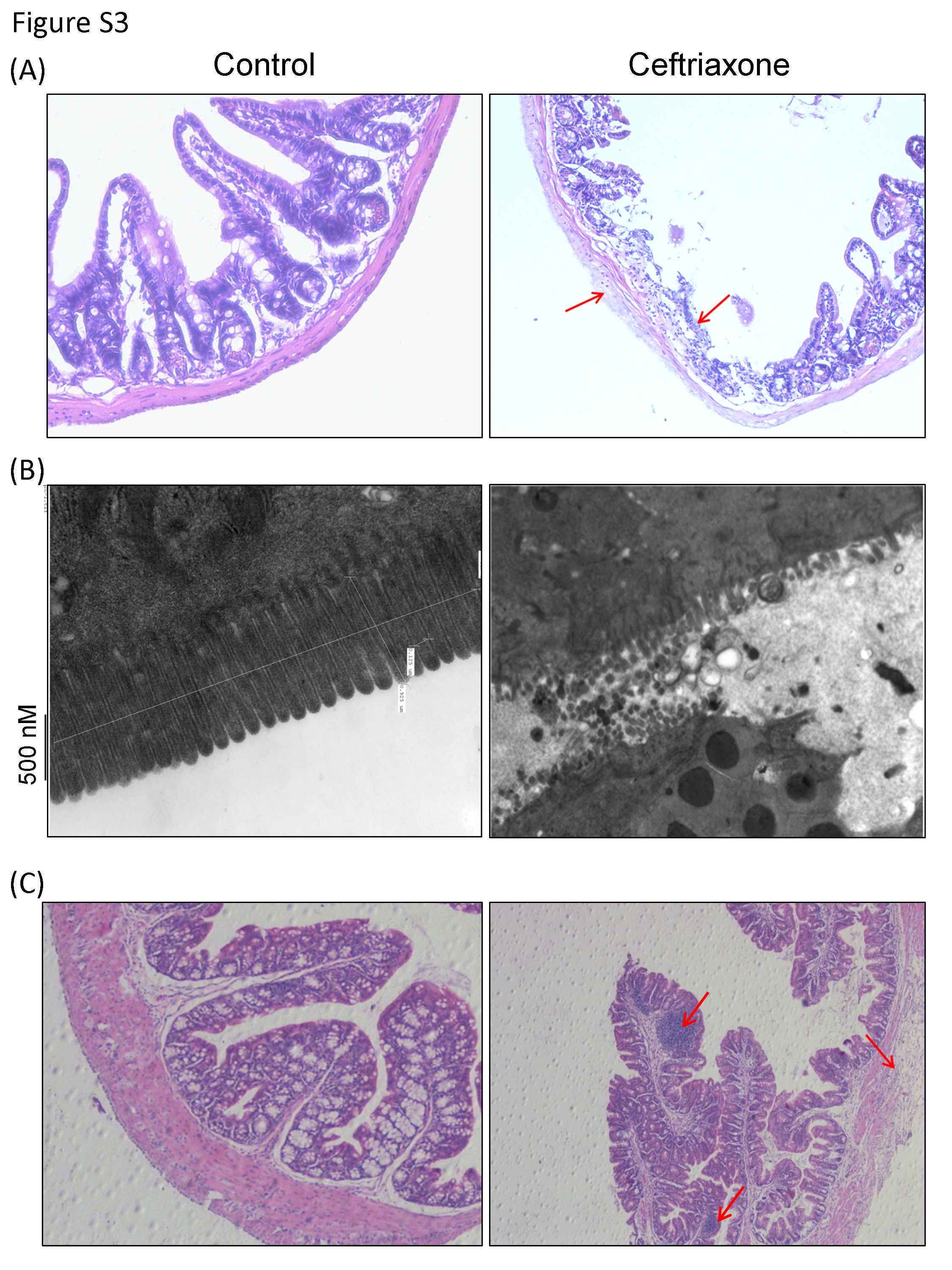

Supplement: Figure S3 — The intestinal phenotype of ceftriaxone-treated mice. (A) Representative patterns of HE-stained sections of distal ileum in mice. Magnification, ×200. (B) Microvilli of ileum in healthy mouse and ceftriaxone-treated mouse. To observe the microvilli, the ultrathin sections were stained with uranyl acetate and lead citrate, and observed under a JEM-1400 TEM (Olympus, Japan). (C) Representative patterns of HE-stained sections of distal ileum in mice. Magnification, ×200. The red arrows indicate inflammatory cell infiltration, edema, or vascular dilatation and congestion. [file Image3.TIF]

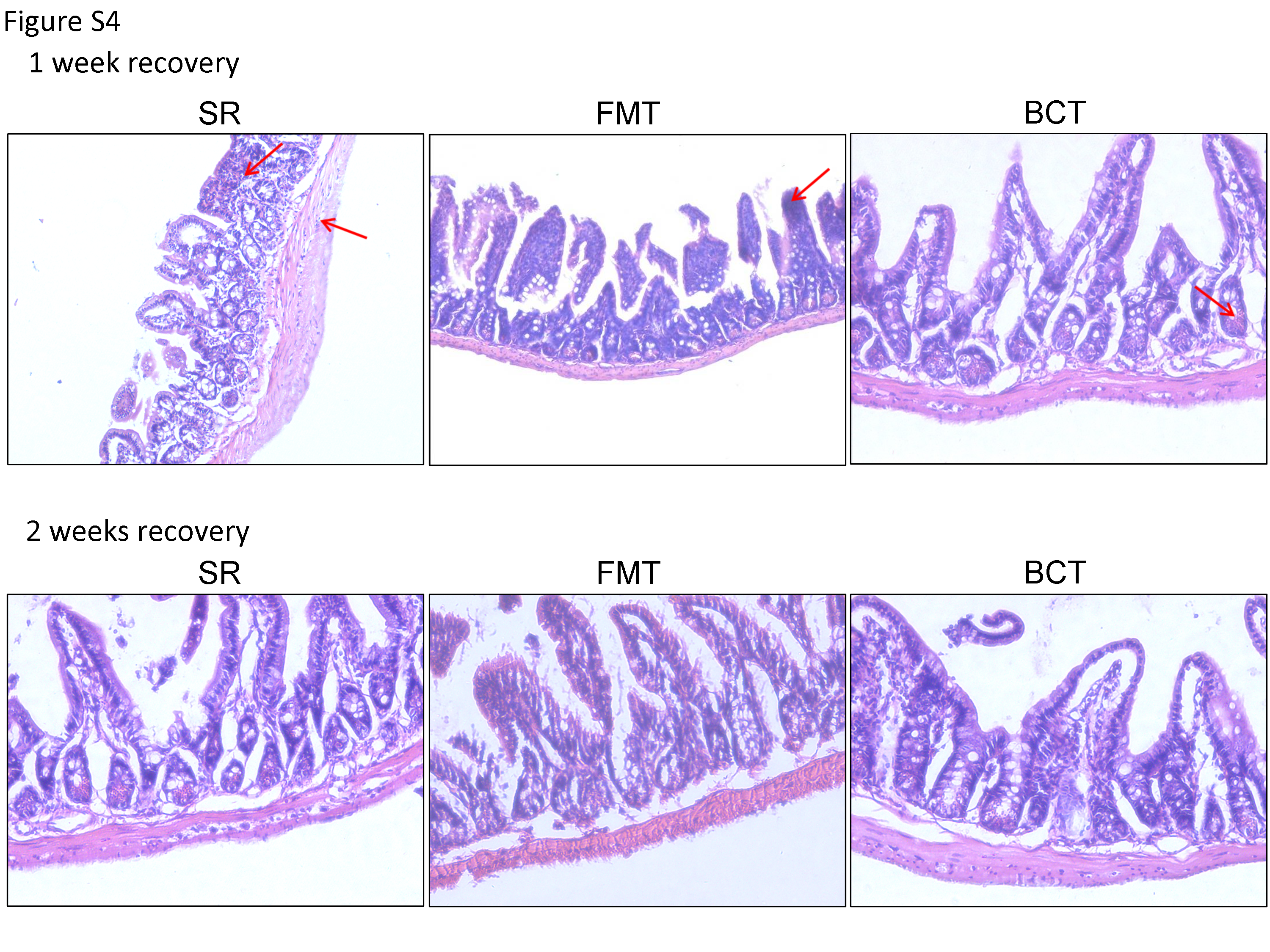

Supplement: Figure S4 — Post-FMT or BCT changes of the ileum in mice. The pictures are representative patterns of HE-stained sections of distal ileum in mice after 1 and 2 week(s) recovery. Magnification, ×200. The red arrows indicate inflammatory cell infiltration, edema, or vascular dilatation and congestion. [file Image4.TIF]

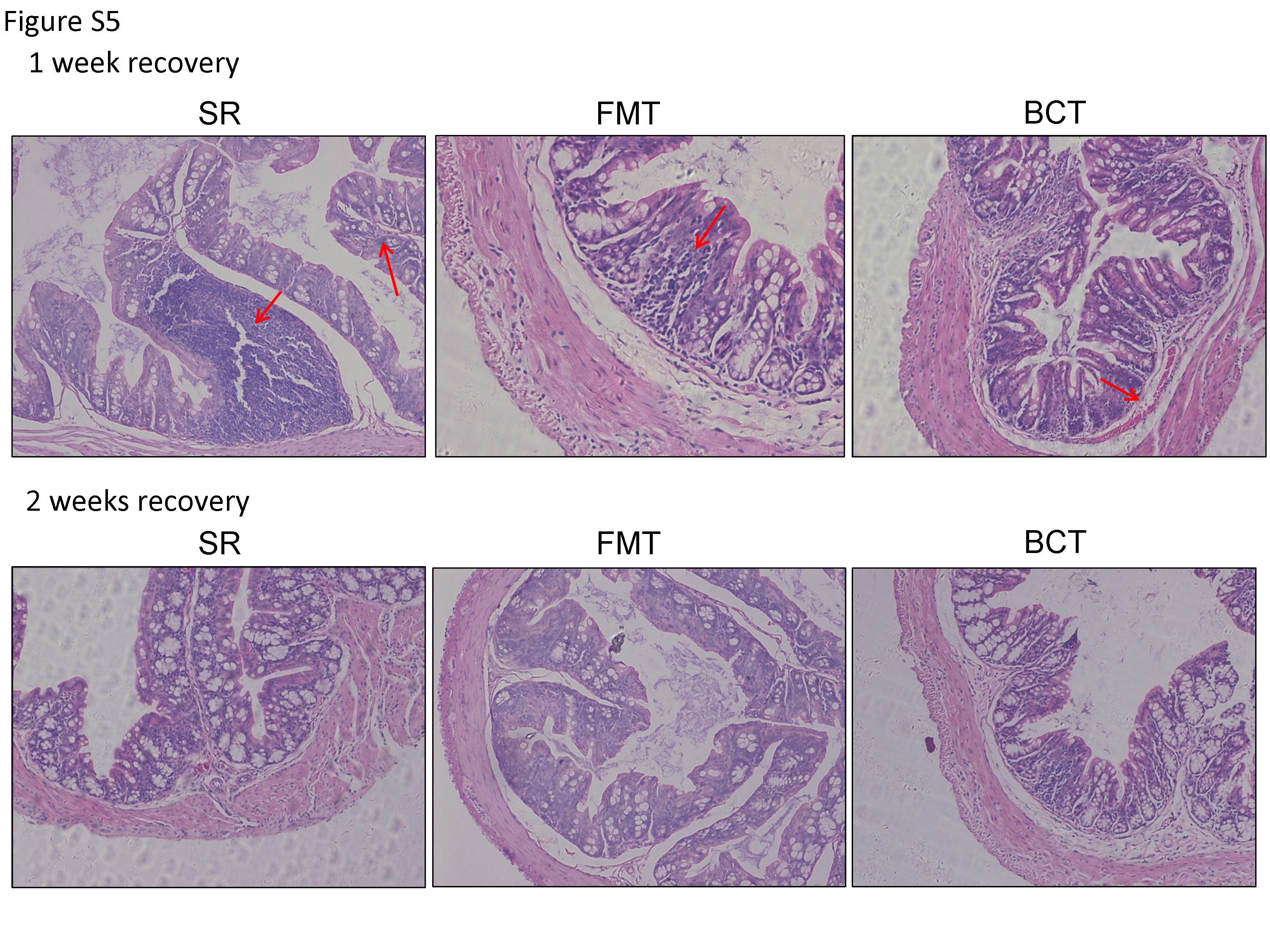

Supplement: Figure S5 — Post-FMT or BCT changes of the colon in mice. The pictures are representative patterns of HE-stained sections of proximal colon in mice after 1 and 2 week(s) recovery. Magnification, ×200. The red arrows indicate inflammatory cell infiltration, edema, or vascular dilatation and congestion. [file Image5.TIF]
